# Supplementary material for: Effectiveness of water-saving technologies during early stages of restoration of endemic Opuntia cacti in the Galápagos Islands, Ecuador
Source: PeerJ. 2019 Dec 3;7:e8156. doi: 10.7717/peerj.8156 (PMC6896940; doi:10.7717/peerj.8156)
Supplement: Appendix S1 — A compilation of photos and methods about Groasis Waterboxx®, and Cocoon system technologies to demonstrate how they look and function in the field. [file peerj-07-8156-s001.pdf]

PLANTING AND MAINTENANCE PROTOCOL FOR GROASIS TECHNOLOGY

INTRODUCTION

Water saving technologies are an efficient tool used in ecological restoration and sustainable agriculture. It makes it possible to plant in areas where water is sparse or costly. In arid regions, the hot climate and strong winds result in the top several meters of soil being very dry. With the water-saving technology "Groasis," primary roots grow up to a centimeter per day. In a year, the roots can achieve a sufficient depth to grow on their own. The technology consists of a conical polypropylene receptacle that collects rainfall and funnels it towards the plant, providing the necessary moisture for the roots to grow (Jaramillo *et al.,* 2015; Hoff, 2014).

BACKGROUND

Galapagos Verde 2050, initiated with a compliance between the Fundación Fuente de Vida (FFV) of Ecuador as a representative of the Dutch organization Groasis, and the Charles Darwin Foundation with the initial funding (Pilot Project) of the COmON foundation. The objective of this agreement was to develop a pilot project for the Groasis Technology in the Galapagos archipelago (Jaramillo *et al*., 2015).

GENERAL OBJECTIVE

Establish the parameters to use the Groasis Technology for ecological restoration and agricultural sustainability. This allows the planting of native and endemic noninvasive species. Furthermore, once established, maintenance is necessary to guarantee results and viability.

METHODOLOGY

STRUCTURAL PARTS OF THE GROASIS TECHNOLOGY

Table 1. The Groasis Technology (Waterboxx) individual structural and functional parts. Adapted from *Waterboxx planting instructions*, October 2019, retrieved from https://es.slideshare.net/pd81xz/yzg14

**Cover:** The cover can be recognized easily because it has a clear color and an oval shaped surface. It is designed to utilize rainfall by draining it into the water box.
**Isolating Plate:** The isolating plate is placed inside the water box (waterboxx) to avoid sun exposure, algae growth, and evaporation.
**Box:** The water box (waterboxx) structure protects the plant and recollects water.
**Wick:** The wick is means to transport the water from the inside of the water box (Waterboxx) to the ground.
**Cardboard plate for evaporation:** The cardboard stock is placed directly in the ground to protect the young plants from dehydration.
**Siphons and cap:** The siphons prevent the water from evaporating, but they allow water to enter the box. The cap closes the Waterbox (Waterboxx).

PRINCIPAL COMPONENTS OF THE GROASIS TECHNOLOGY

Table 2. Components of the Groasis Technology. Adapted from *Waterboxx planting instructions*, October 2019, retrieved from https://es.slideshare.net/pd81xz/yzg14 and from *Ten times re-usable Waterboxx® plant cocoon*, October 2019, retrieved from https://www.groasis.com/

**Groasis Technology**	
**Material**	Virgin polypropylene
**Total Weight**	1.4 kg (kilograms)
**Componentes**	Resevoir water box
	Isolating plate (black lid)
	2 siphons
	1 or 2 wicks
	A cover for collecting rainfall
	A blue cap
**Extra Parts**	Wind protectors and anchoring system
**Capacity**	20 liters of water
**Model**	Reusable
**Toxicity**	Null
**Storage**	Fresh areas

MATERIALS AND EQUIPMENT

- ✓ Shovel
- ✓ Rake
- ✓ Gloves
- ✓ Container with 20 L of water
- ✓ Cardboard plate (white)
- ✓ Water box:
    - Base of the box (Green)
    - Wick
    - Tube of silicon
    - Isolating plate (black)
    - Cover (White)
    - 2 siphons (Blue)

- ✓ Growsafe
- ✓ Aluminum protective mesh

PROCESS

The methodology of planting with Groasis Technology is composed of the following steps (Groasis, 2019):

Preparation of soil

The first step is to prepare the soil with or without compost and place the Waterboxx into the ground. Keep in mind the ground preparation has to be executed 24 hours before planting takes place.

When should compost be considered?

When the soil is very unhealthy or has a hard time retaining water. For example, if the ground is mushy or loose. The usage of fertilizer creates a proportional balance of nutrients for the plant and avoids water from disappearing directly from the ground.

Dig a hole

Dig a hole approximately 15 cm deep and 60 cm wide. The base must be even and free from rocks to place the Waterboxx firmly in the ground. Place 5 to 10 Liters of water in the hole, the amount of water depends on the soil type. The water will be transferred in a container of water in case there is no supply at the sowing point (Figure 1).

- Pour water directly in the hole and wait 24 hours before planting

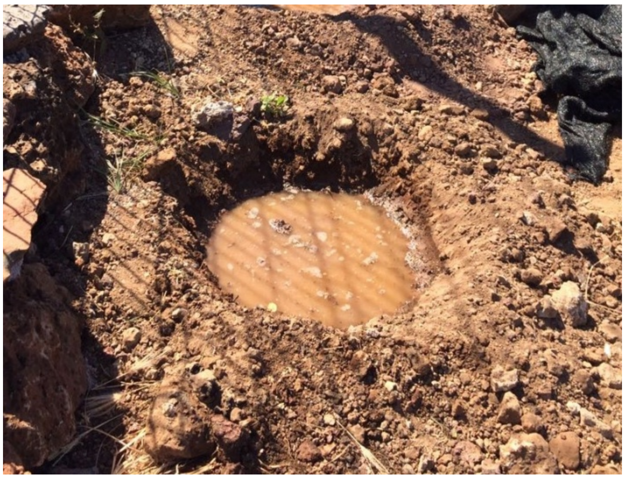

Figure 1. Ground prepared for planting

- Plant the seedling from the nursery, removing the plastic cover. The roots of the seedling should be carefully arranged in a vertical position. The base of the seedling should be fixed with soil to ensure its stability over time and erect growth (Figures 2 & 3).

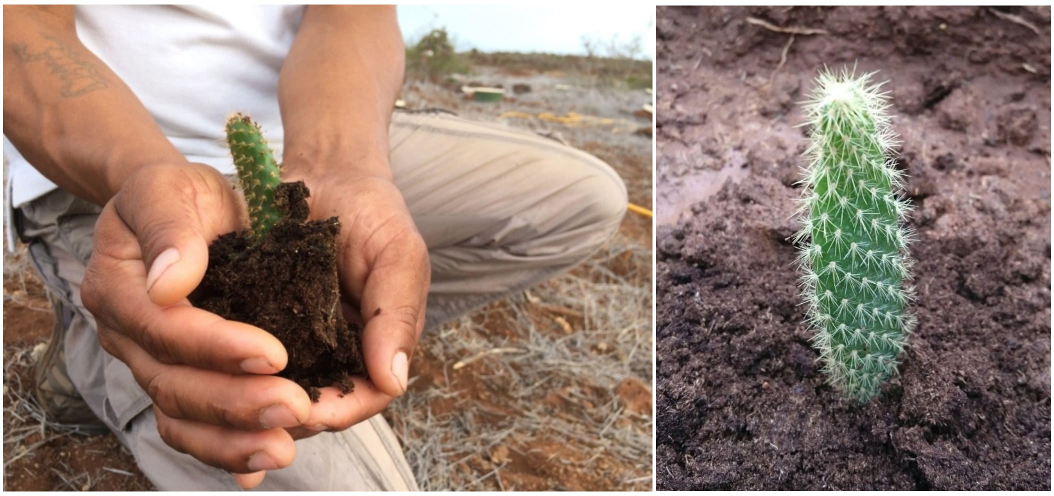

Figures 2 & 3. Planting a species of *Optunia*

- Place the white cardboard plate on the moist ground, ensuring that the indication of north on the plate goes towards the geographic north. This address minimizes hours of exposure to direct sunlight (Figure 4).

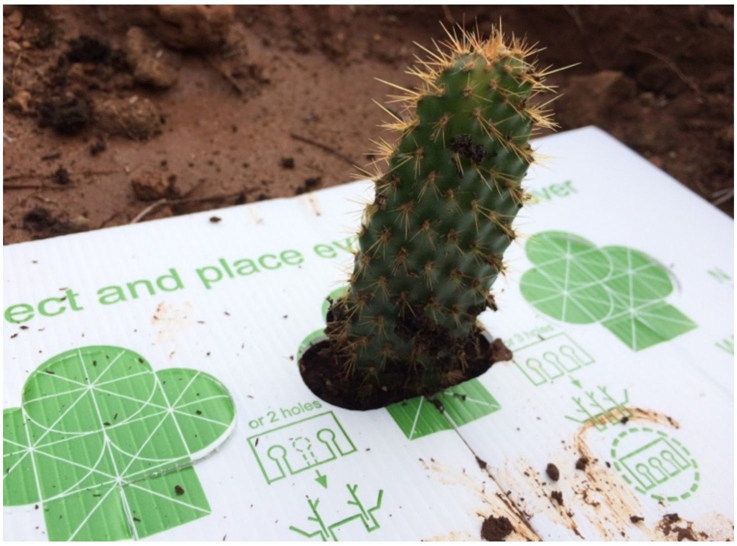

Figure 4. Plate facing north

- Place the water box (Waterboxx) with the wick placed underneath the cardboard to have a direct transportation of water to the root of the plant. The box is placed gently to ensure no damage to the plant. Furthermore, the plant must not be in the center of the box as certain plants may not have sufficent space. The white cover will help with the cardinal directions (Figure 5).

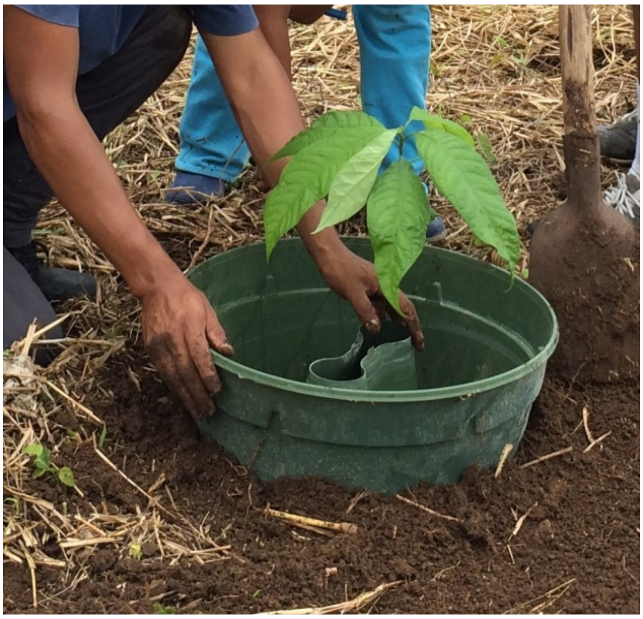

Figure 5. Placing a Groasis box over a *Scalesia* plant

- Pour 10 L of water into the water box. After the planting process the box is left with that water supply. If rain conditions allow it, it will not be necessary to re-supply water later (Figure 6).

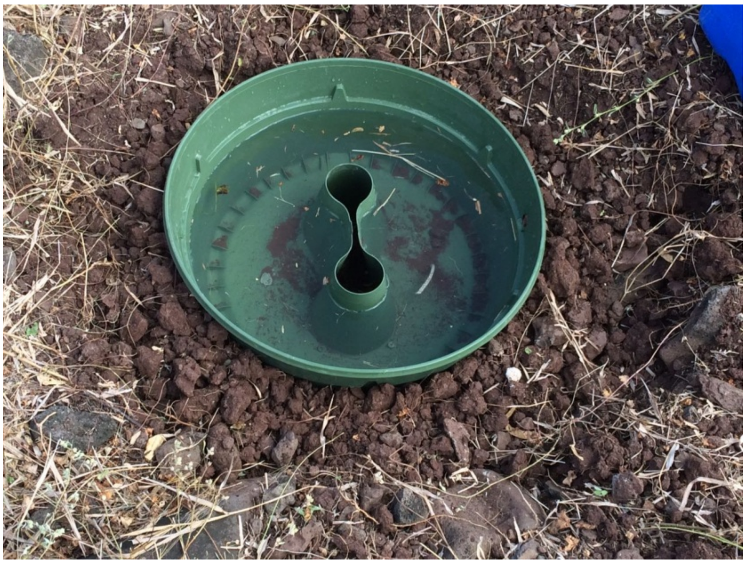

Figure 6. Groasis box with water

- Placement of the isolating plate. This plate allows the water that evaporates to be retained in the box, where it condenses and falls back inside the box, minimizing the loss of water (Figure 7).

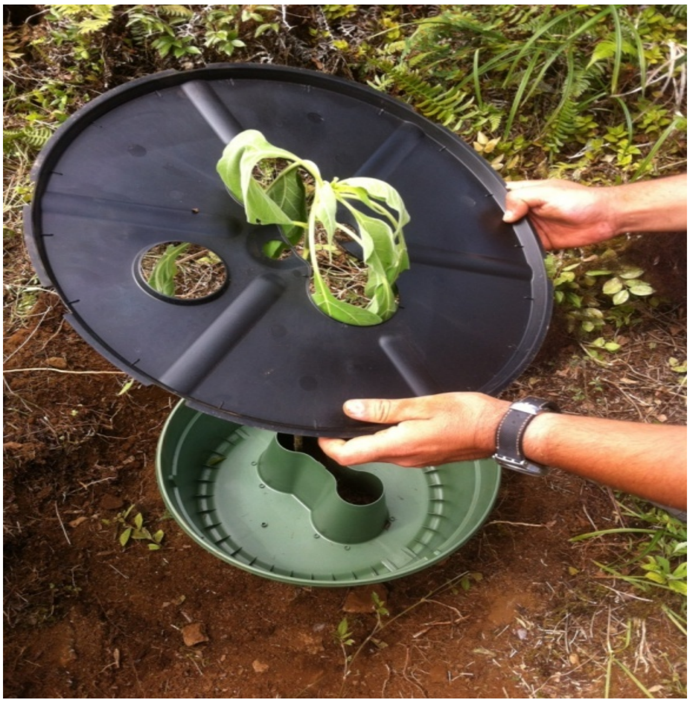

Figure 7. Isolating plate on the seedling

- Placement of the cover and two siphons. The tabs of the box must fit correctly with the corresponding holes in the white cover. The cover protects the seedling from attacks that could be suffered by animals or high temperatures and direct sun exposure (Figures 8, 9 and 10).

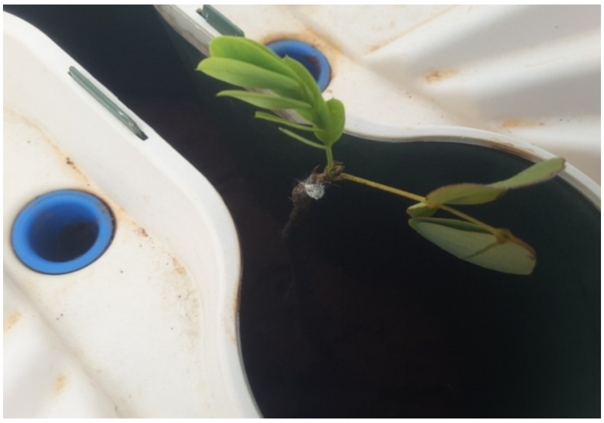

Figure 8. Aligning the tabs of the box with the holes in the cover

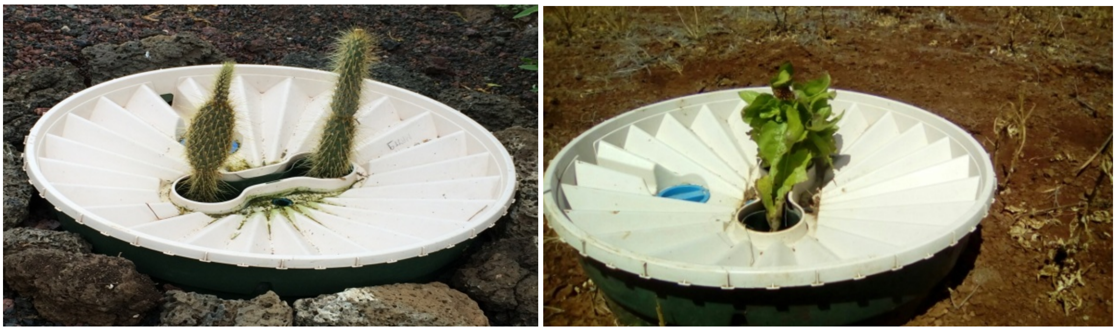

Figures 9 & 10. Final assembly of the Groasis Waterboxx with the cover and siphons

- Place plastic Growsafe protector and the metallic mesh, in the appropriate cases, to avoid the attack of animals and minimize direct sunlight (Figures 11 and 12).

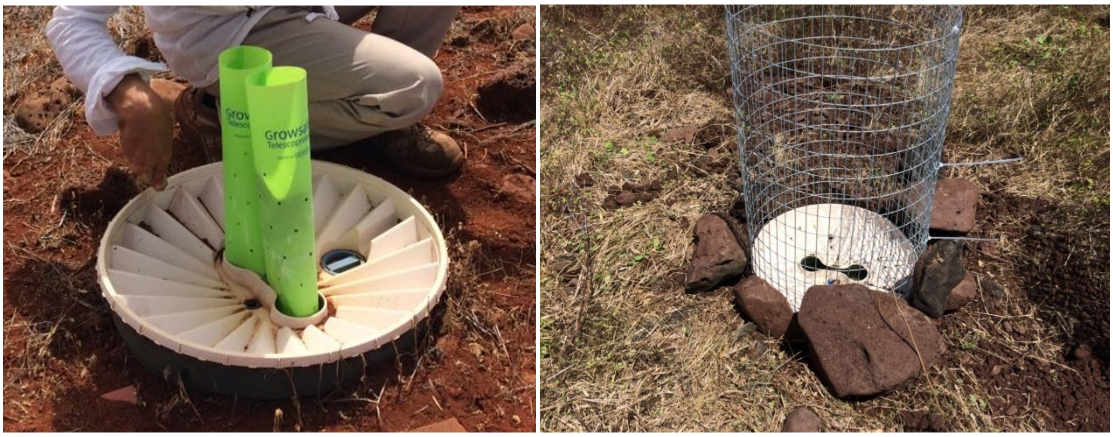

Figures 11 & 12. Growsafe and aluminum mesh for animal attack protection

- Mark the boxes and controls with a unique numerical code for each plant. As a control 20% of the total number of plants will be planted without the technology.
- Record environmental and biological information of each individual.
- Remove Groasis Box once the plant reaches sufficient size to develop independently and reuse the box (Figures 13, 14, 15 and 16).

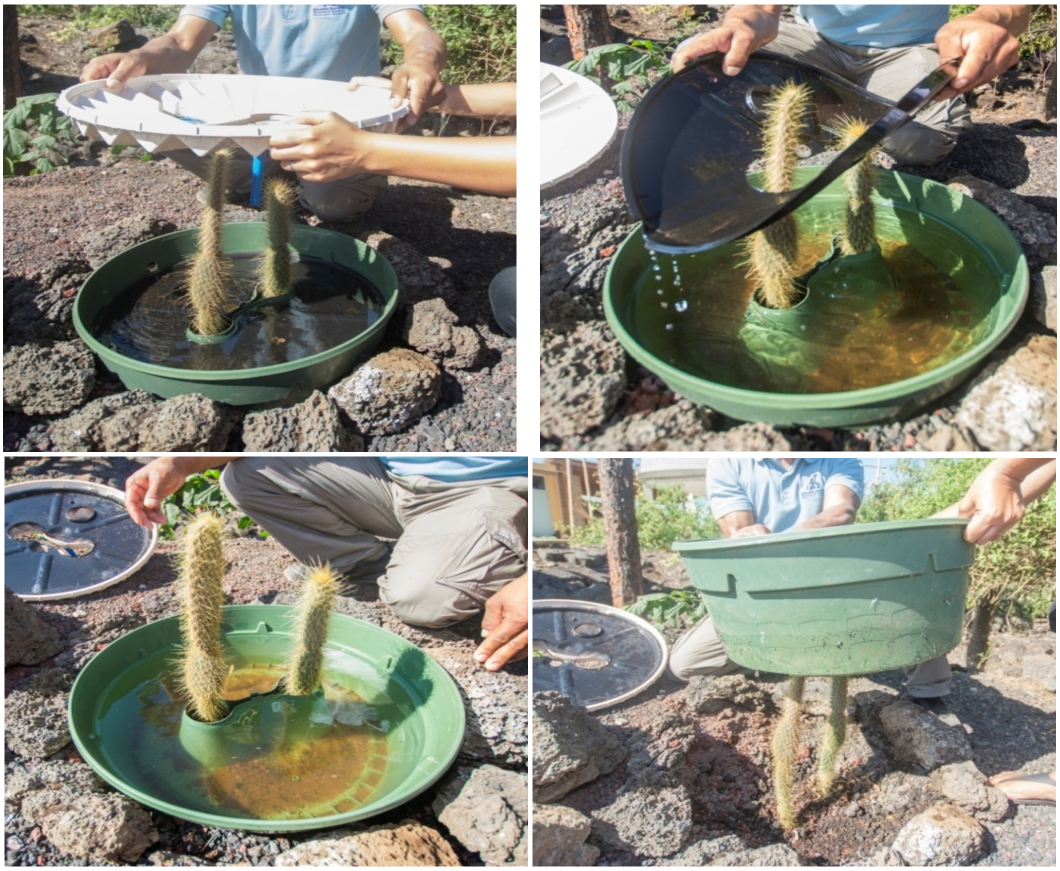

Figures 13, 14, 15 & 16. Process of removing the Groasis Technology

- Once the Groasis Technology has been removed, the plant is ready to survive on its own. If two plants were placed at the same time then one of them must be transplanted to another place, because it is the right time to do so (Figures 17 and 18).

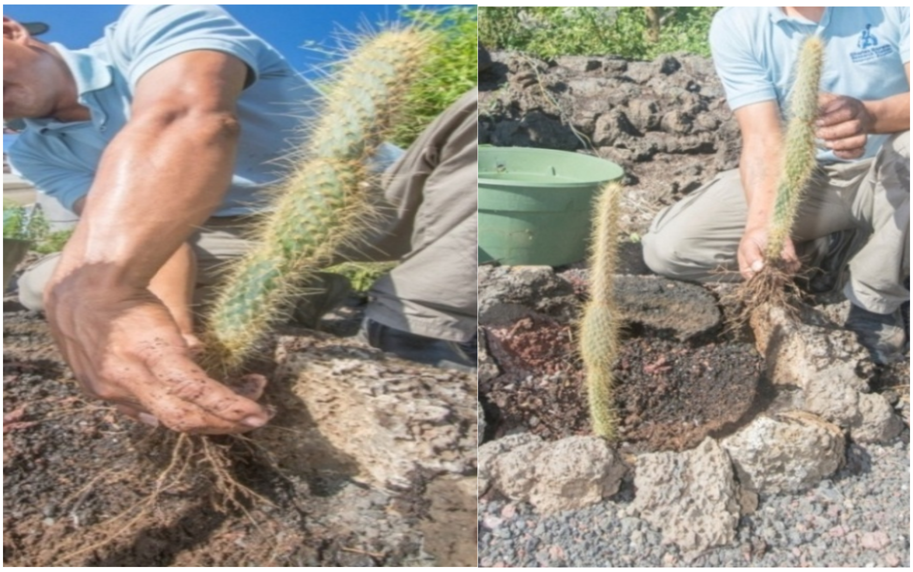

Figures 17 & 18. Transplanting one of the two *Opuntias* that were planted with the Groasis Technology

Maintenance of Groasis Technology

The plants that have been planted are monitored roughly every three months. During the monitoring routes there is maintenance of the boxes to be done by the following criteria (Figures 19 & 20):

- Checking the Growsafe to ensure it is in good condition. If it is in poor condition, it is substituted with a new one.
- The codes are checked to ensure that they are legible. If they are not they must be rewritten.
- The next step is to lift the cover carefully in order not to damage the plant. Using a brush, clean the residue of organic matter that may have built on the siphons.
- Clean the siphons with a long stick to remove any soil that may be blocking them. The siphons' task is to drain rainwater into the box.
- If the box does not have water and the plant needs it, it will be provided with extra water.

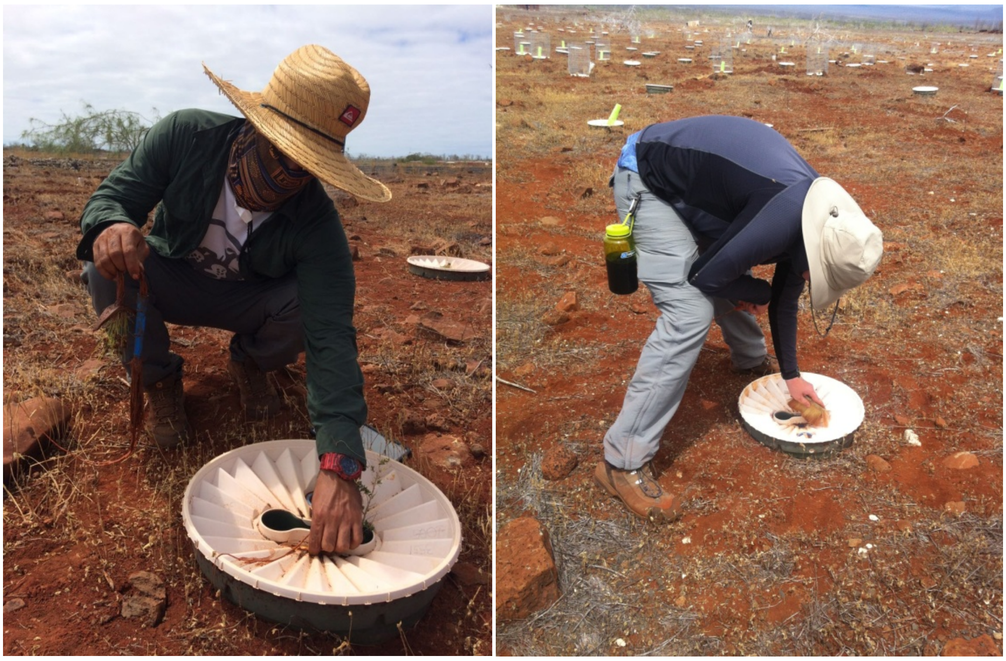

Figures 19 & 20. Siphons and Groasis cover being cleaned

PROTOCOL FOR PLANTING WITH COCOON TECHNOLOGY

INTRODUCTION

Cocoon Technology is 100% degradable. It is composed of paper, cardboard, nylon, and wax. It is designed to support a young plant by providing water and shelter while it stimulates deep and healthy roots during their first year; which is critical (Land Life Company, 2019).

BACKGROUND

The water saving technology testing went so well that in 2014 the Galapagos Verde 2050 team initiated testing the biodegradable Cocoon on Baltra Island.

The study started on Baltra Island in the three main study sites and in the gardens of the CDF and PNG.

GENERAL OBJECTIVE

- Establish the parameters that must be followed when planting the native, endemic and noninvasive cultivated species, both in the ecological restoration component and in sustainable agriculture using the water-saving Cocoon Technology.

METHODOLOGY

COMPONENTS OF THE COCOON TECHNOLOGY

Table 3. Components of Cocoon Technology. Adapted from *A new way to fix the planet*, October 2019, retrieved from https://landlifecompany.com/technology/

Cocoon Technology	
**Materials**	Cellulose
	Crop or pasture residue
	Organic wax for impermiability
**Total Weight**	1.1 kg
**Components**	Water reservoir box
	Lid to the reservoir
	Wind protector
	2 wicks
**Capacidad**	25 liters of water
**Degradabilidad**	100% biodegradable
**Toxicidad**	Null
**Almacenamiento**	Fresh areas, without humidity or sun exposure

MATERIALS AND EQUIPEMENT

- ✓ Shovel
- ✓ Rake
- ✓ Gloves
- ✓ 20 L of water
- ✓ Cocoon box
- ✓ Wick

PROCESS

The methodology of planting with Cocoon Technology is composed of the following steps (Land Life Company, 2019):

- Dig a hole in the chosen location that is 15 cm deep. The ground must be leveled and free from stones. Pour 5 to 10 L of water in the hole, depending on soil type. In case of not having a water supply near the planting site, you may be required to transport water in a container (Figure 21).

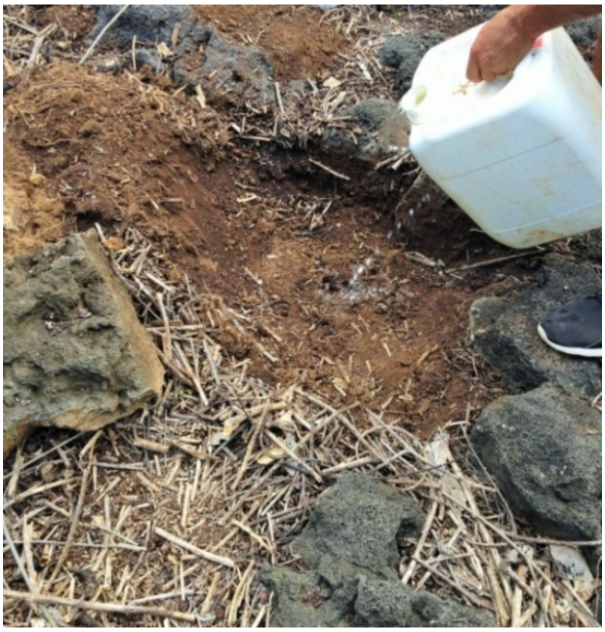

Figure 21. Preparing the ground for planting

- Plant the seedling from the nursery, removing the plastic cover. The roots of the seedling should be carefully arranged in a vertical position. The seedling base should be fixed with soil to ensure its stability over time and allow upright growth (Figure 22).

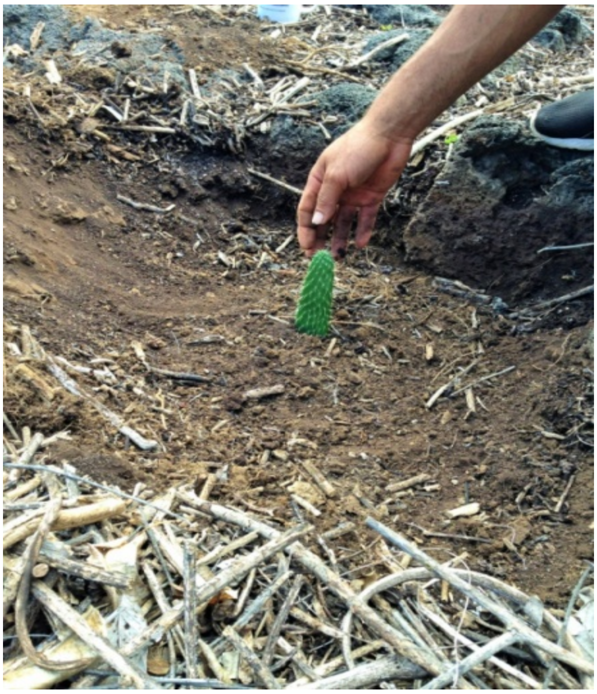

Figure 22. Planting the *Optunia* seedling

- Place the Cocoon box over the plant and fill with water (Figures 23 & 24).

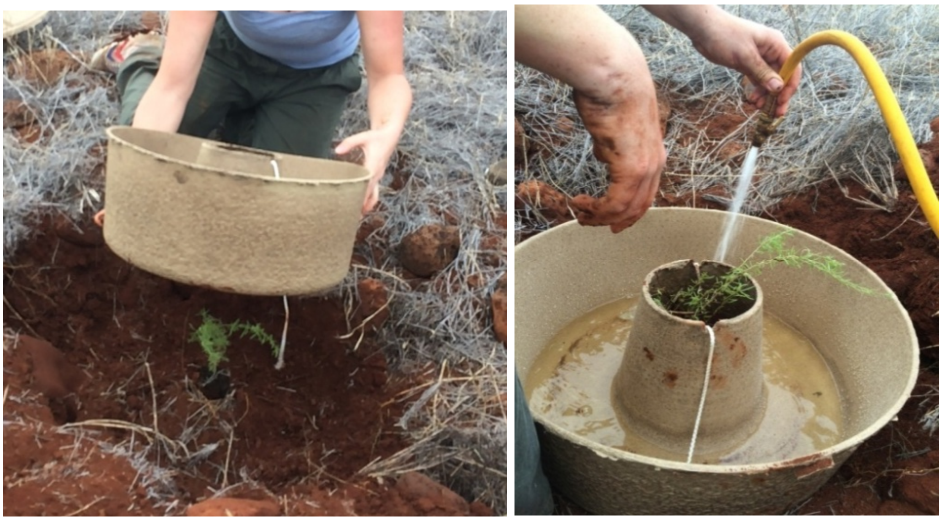

Figures 23 & 24. Placing the Cocoon box over plants and adding water

- Place the wick near the plant to allow water to be transported (Figures 25 & 26)

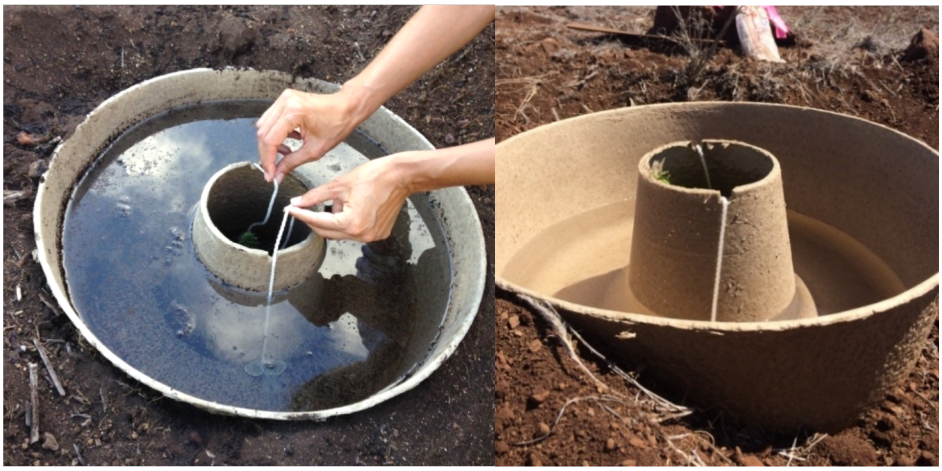

Figures 25 & 26. Correctly placing the wick towards the plant

- Place the lid and the card board protector. The protector minimizes attacks from animals and direct sunlight exposure (Figures 27 & 28).

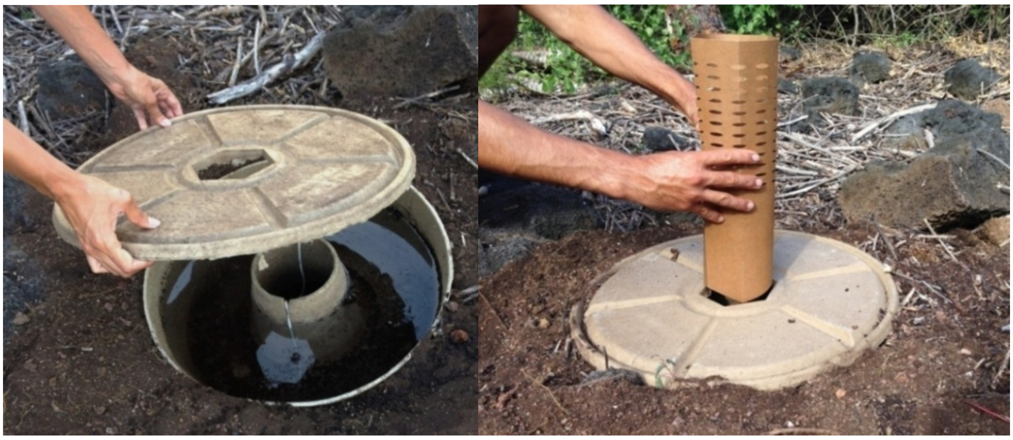

Figures 27 & 28. Lid and protector being placed for protection

- Cover the Cocoon with soil for it to remain buried (Figure 29).

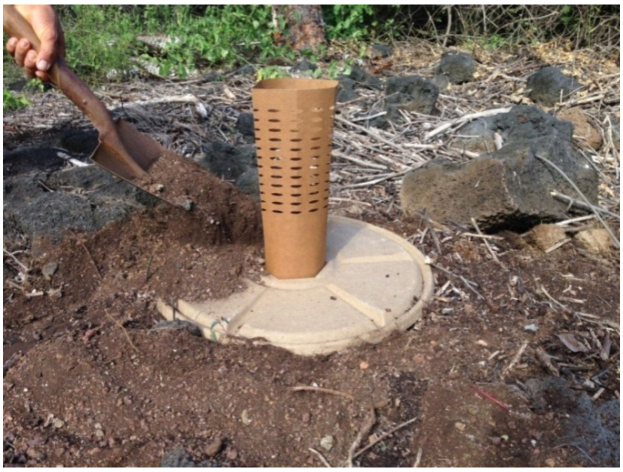

Figure 29. Cocoon being covered with soil to preserve and protect it

- Once planting with Cocoon Technology is finished, it will not require maintenance and will degrade within two years. As a control, 20% of the total plants must be planted without technology. The Cocoon Technology is biodegradable so there is no requirement to remove it (Figures 30 & 31).

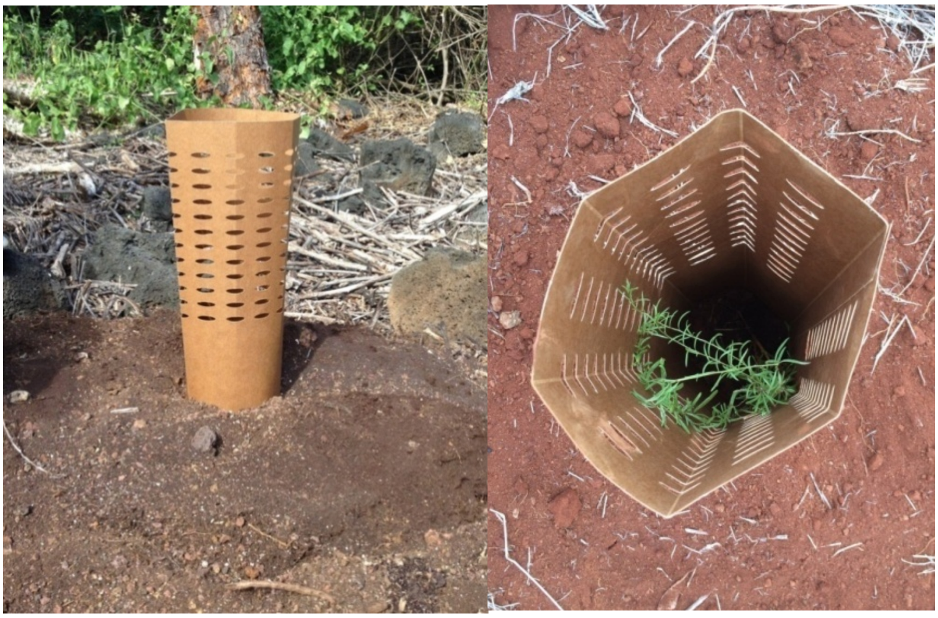

Figures 30 & 31. The Cocoon Technology is biodegradable so there is no requirement to remove it

References in addition to main manuscript

Groasis®. (2019). Waterboxx® plant cocoon. Retrieved from https://www.groasis.com/en

Hoff, P. (2014). Groasis Technology: Manual de Instrucciones de plantación: 1-27.

Jaramillo, P., Lorenz, S., Ortiz, G., Ortiz, J., Rueda, D., Gibbs, J.P., & Tapia, W. (2015). Galápagos Verde 2050: An opportunity to restore degraded ecosystems and promote sustainable agriculture in the Archipelago. Biodiversity and Ecosystem Restoration: GNPD, GCREG, CDF, and GC. In Cayot, L., Cruz, D. & Knab, R. (ed.). *Galápagos Report 2013-2014*, pp. 133-143.

Land Life Company. (2019). A new way to fix the planet. Retrieved from https://landlifecompany.com/technology/
