# Supplementary figures and images for: Effectiveness of water-saving technologies during early stages of restoration of endemic Opuntia cacti in the Galápagos Islands, Ecuador
Source: PeerJ. 2019 Dec 3;7:e8156. doi: 10.7717/peerj.8156 (PMC6896940; doi:10.7717/peerj.8156)

# Total Rainfall each month (2014-2018)

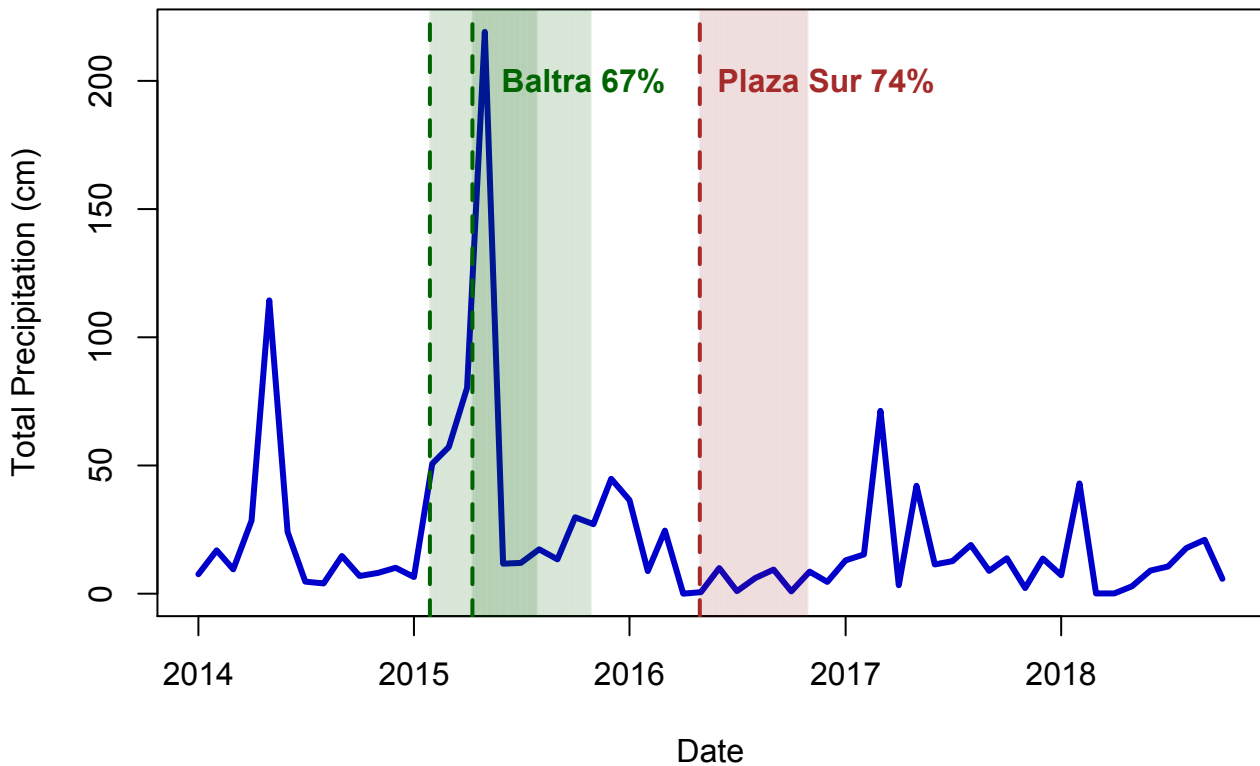

Supplement: Appendix S2 — Dotted lines indicate the date(s) when the majority of plantings occurred on Baltra (67%) and Plaza Sur (73%). Only Baltra and Plaza Sur are included since these two islands made up the majority ofOpuntia plantings considered in this study (92%). Shaded areas indicate the six-month period following plantings, showing that 73% of Plaza Sur plantings occured just before the longest period of reduced precipitation between 2014 and 2018. [file peerj-07-8156-s002.pdf]
